# Phytase Modulates Ileal Microbiota and Enhances Growth Performance of the Broiler Chickens

**DOI:** 10.1371/journal.pone.0119770

**Published:** 2015-03-17

**Authors:** Anna Ptak, Michael R. Bedford, Sylwester Świątkiewicz, Krzysztof Żyła, Damian Józefiak

**Affiliations:** 1 PIAST GROUP Research and Development Center, Ostrów Wielkopolski, Poland; 2 AB Vista Feed Ingredients, Marlborough, United Kingdom; 3 Department of Animal Nutrition and Feed Science, National Research Institute of Animal Production, Balice, Poland; 4 Department of Food Biotechnology, University of Agriculture, Kraków, Poland; 5 Department of Animal Nutrition and Feed Management, Poznań University of Life Sciences, Poznań, Poland; University of New England, AUSTRALIA

## Abstract

Phytase is well studied and explored, however, little is known about its effects on the microbial ecology of the gastrointestinal tract. In total, 400 one-day-old female Ross 308 chicks were randomly distributed to four experimental groups. The dietary treatments were arranged as a 2 × 2 complete factorial design, with the factors being adequate (PC) or insufficient calcium (Ca) and digestible phosphor (dP)(NC) and with or without 5000 phytase units (FTU)/kg of *Escherichia coli* 6-phytase. The gastrointestinal tract pH values, ileal microbial communities and short-chain fatty acid concentrations in the digesta were determined. The reduction in Ca and dP concentration significantly affected pH in the crop and caeca, and addition of phytase to the NC resulted in a pH increase in the ileum. The reduction in Ca and dP concentration significantly lowered, while phytase supplementation increased ileal total bacterial counts. Additionally, the deficient diet reduced butyrate- but increased lactate-producing bacteria. The addition of phytase increased *Lactobacillus sp*./*Enterococcus sp*. whereas in case of *Clostridium leptum* subgroup, *Clostridium coccoides* - *Eubacterium rectale* cluster, *Bifidobacterium sp*. and *Streptococcus/Lactococcus* counts, a significant Ca and dP level x phytase interaction was found. However, the recorded interactions indicated that the effects of phytase and Ca and dP levels were not consistent. Furthermore, the reduction of Ca and dP level lowered *Clostridium perfringens* and *Enterobacteriaceae* counts. The analysis of fermentation products showed that reducing the Ca and dP content in the diet reduced total SCFA, DL-lactate, and acetic acid in the ileum whereas phytase increased concentrations of these acids in the NC group. This suggests that P is a factor which limits fermentation in the ileum. It may be concluded that phytase plays a role in modulating the gut microbiota of chicken, however, this is clearly linked with the levels of P and Ca in a diet.

## Introduction

Phytase is probably one of the most studied and explored exogenous enzymes used in non-ruminant nutrition. Its effect on performance, nutrient retention and availability of many nutrients including the macroelements and trace minerals has been documented in many studies in different animal species [1–6].

The incorporation of the phytase into the matrix of poultry diets leads to significant changes in the utilization of dietary limestone and phosphates. Through P and Ca release from phytate complexes, phytase reduces the amount of inorganic phosphate and calcium required in feed formulation, which in most cases is provided in the form of mono- or dicalcium phosphates. However, because phytase releases more P than Ca (as a proportion of the requirement), its application results in additional limestone being added to maintain an approximate 2:1 Ca:P ratio in the diet. This likely may change the physical and chemical properties of the digesta in the gastrointestinal tract. Shafey et al. [7] reported that increasing dietary calcium from 1.07 to 2.53% increased crop pH from 4.89 to 5.32 and in the ileum from 6.62 to 7.39. More recently Walk et al. [8] showed that increasing Ca concentration from 0.64 to 1.03% elevated pH in the gizzard by 0.15. McDonald and Solvyns [9] increased digesta pH from 5.6 to 6.1 along the whole length of the small intestine by increasing the level of dietary calcium, and this measure was negatively correlated with broiler chicken performance. An excess in buffering capacity may interfere with digestion rate and extent, particularly during the gastric phase, and may also force additional commitment of digestive resources (hydrochloric acid and pepsin) if digestion is to be maintained. If gastric digestive capacity is exceeded then there is a risk that some protein may resist digestion and be fermented in the large intestine which may lead to poor performance and increase the risk of intestinal disease [10, 11].

Changes in the digesta pH may result in shifts of endogenous microbiota profiles and in their activity. Moreover, it is considered that a lower pH in the small intestine, due to its bacteriostatic effect, should be positive for intestinal integrity and microecology by reducing the occurrence of potentially pathogenic *Enterobacteriaceae* and increasing favourable lactic acid bacteria (LAB) populations [12]. Therefore, the effect of phytase observed in many studies could have been related to a reduction in the buffering capacity of a diet with subsequent effects on microbiota profiles. However, in the available literature very little data are available on the direct effect of phytase on the status of the microbiome of the host.

The aim of the present study was to investigate effects of supplemental phytase on the performance of broiler chickens fed diets with different Ca and dP levels and on microbiota populations and their metabolites, in different parts of the gastrointestinal tract.

## Material and Methods

### Ethics Statement

This study was carried out in strict accordance with the recommendations of the National Ethic Commission (Warsaw, Poland). All procedures and experiments complied with the guidelines and were approved by the Local Ethic Commission of the Poznań University of Life Sciences (Poznań, Poland) with respect to animal experimentation and care of animals under study, and all efforts were made to minimize suffering.

### Birds and Housing

In total, 400 one-day-old female Ross 308 chicks, obtained from a commercial hatchery, were randomly distributed to 4 experimental groups using 10 replicate pens per treatment and 10 birds per pen. The broiler chickens were kept in floor pens (1 × 1 m) arranged by block in the center of a commercial chicken house to 42 days (d) of age. To simulate commercial production conditions, the experimental pens were surrounded by a commercial broiler flock composed of birds of the same origin as those used in the experiments. All pens were of the same dimensions and had the same number of nipple drinkers and feed hoppers. The birds were given 23 h of light and 1 h of dark during the first week and then 19 h of light and 5 h of dark from d 7 to 21. From 22 to 42 d of age, there was 23 h of light and 1 h of dark.

### Diets and Feeding Program

The composition of the experimental diet is shown in Table 1. Diets for each period were formulated to be isonitrogenous and isocaloric. The diets were prepared in mash form; all raw materials were ground by disc mill (Skiold A/S, Denmark) at 2.5 mm disc distance, mixed without any heat treatment, and fed *ad libitum* to the birds. Starter diets were offered to all birds from 1 until 14 d of age and finisher diets from 15 to 42 d of age. The dietary treatments were arranged as a 2 × 2 complete factorial design, with the factors being adequate or insufficient Ca and digestible P and with or without 5000 phytase units (FTU)/kg of *Escherichia coli* 6-phytase, EC 3.2.1.26, produced in *Pichia pastoris* DSM 15927 – Quantum 2500 (AB Vista Feed Ingredients, Marlborough, UK).

**Table 1:** Composition of the basal diets and its calculated nutritive value.

	Starter diet (1–14d)		Finisher diet (15–42d)	
Ingredients (g/kg)	Positive control^a^	Negative control	Positive control	Negative control
Wheat	66.71	68.17	66.75	68.21
Rapeseed expeller	5.00	5.00	10.00	10.00
Soybean meal	22.19	21.91	15.29	14.02
Soybean oil	2.27	1.84	4.91	4.48
Dicalcium phosphate	1.78	0.81	1.20	0.24
Limestone	0.63	0.83	0.56	0.76
Salt (NaCl)	0.29	0.29	0.21	0.21
Sodium carbonate (Na_2_CO_3_)	0.08	0.08	0.11	0.12
L-Lizyna HCl	0.41	0.42	0.39	0.39
Methionine hydroxy analogue	0.27	0.27	0.25	0.24
L-Threonine	0.09	0.08	0.05	0.05
Vitamin-mineral premix^b^	0.30	0.30	0.30	0.30
Calculated nutritive value				
AMEn, kcal/kg	2900	2900	3100	3100
Crude protein, %	21.44	21.51	19.88	19.95
Crude fat, %	3.97	3.56	6.86	6.46
Sodium-total, %	0.16	0.16	0.14	0.14
Calcium-total, %	0.85	0.71	0.70	0.56
Phosphorus-total, %	0.72	0.55	0.62	0.46
P-digestible, g/kg	4.00	2.69	3.20	1.89
Lysine, %	1.27	1.28	1.15	1.15
Methionine, %	0.54	0.54	0.51	0.51
Methionine + Cystine, %	0.93	0.93	0.89	0.89
Threonine, %	0.81	0.81	0.72	0.72

^a^Positive control diet adequate in P and Ca; negative control diet with dP and Ca levels reduced by 0.13 and 0.14%, respectively. dP = digestible P.

^b^Provided the following per kilogram of diet: vitamin A, 11,166 IU; cholecalciferol, 2,500 IU; vitamin E, 80 mg; menadione, 2.50 mg; vitamin B12, 0.02 mg; folic acid, 1.17 mg; choline, 379 mg; d-pantothenic acid, 12.50 mg; riboflavin, 7.0 mg; niacin, 41.67 mg; thiamine, 2.17 mg; d-biotin, 0.18 mg; pyridoxine, 4.0 mg; ethoxyquin, 0.09 mg; Mn (MnO_2_), 73 mg; Zn (ZnO), 55 mg; Fe (FeSO_4_), 45 mg; Cu (CuSO_4_), 20 mg; I (CaI_2_O_6_), 0.62 mg; and Se (Na_2_SeO_3_), 0.3 mg.

### Data and Sample Collection

The feed intake and body weight of the chickens were measured on days 14, 21 and 42. Mortality was registered throughout the entire experiment. At the end of the trial (42 d), 20 randomly picked chickens (2 chickens from each of the 10 replicate pens) from each experimental group were killed by cervical dislocation. For analyses of gastrointestinal content pH, the contents of crop, ileum and caeca from 2 birds per pen were pooled (10 replicate digesta samples of approx. 10g). The pH in these combined samples was measured immediately after slaughter using a combined glass and reference electrode. The remaining portion of the combined samples were immediately frozen and stored in −80°C for the analysis of organic acids by gas chromatography and the microbiota composition by fluorescent *in situ* hybridization of single bacterial cells (FISH).

### Microbiota Analysis by Fluorescent In Situ Hybridization

For FISH analysis, 100 μL of the ileal digesta were diluted in PBS and pipetted onto 0.22 μm polycarbonate filters (Frisenette K02BP02500) and vacuumed (Vaccum KNF Vacuport-Neuberg). After vacuuming, the filters were transferred onto cellulose discs for dehydration in an ethanol series (50, 80, and 96%, 3 min. each). For each sample, a series of identical filters were prepared to allow the determination of optimal hybridization [13, 14]. The oligonucleotides probes used for this study (Table 2) were selected from the literature. Hybridizations were carried out in 50 μL of hybridization buffer (0.9 *M* NaCl; 20 m*M* Tris/HCl, pH 7.2; 0.01% SDS) containing the oligonucleotides probes (Table 2). After hybridization, the filters were washed with washing buffer (20 m*M* Tris/HCl, pH 7.2; 0.01% SDS; 5 m*M* EDTA) for 20 min. at 48°C. The filters were rinsed gently in distilled water, air-dried, and mounted on object glasses with VectaShield (Vector laboratories nr. H-1000) anti-fading agent containing DAPI (4',6-diamidino-2-phenylindole) as described in details by Józefiak et. al. [15]. The area of each filter was 255 mm^2^.

**Table 2:** Probes used in the determination of ileal microbiota by *in situ* fluorescent hybridization (FISH).

Target	Probe	Sequence (5' to 3')
*Bacteroides*-Prevotella cluster	Bac303	CCAATGTGGGGGACCTT^**1**^
*Clostridium coccoides*—*Eubacterium rectale* cluster	Erec482	GCTTCTTAGTCARGTACCG^**2**^
*Enterobacteriaceae*	Enter1432	CTTTTGCAACCCACT^**3**^
*Clostridium leptum* subgroup	Clept1240	GTTTTRTCAACGGCAGTC^**4**^
*Streptococcus/Lactococcus*	Strc493	GTTAGCCGTCCCTTTCTGG^**2**^
*Bifidobacterium sp*.	Bif228	GATAGGGACGCGACCCCAT^**5**^
*Lactobacillus sp*./*Enterococcus sp*.	Lab158	GGTATTAGCAYCTGTTTCCA^**6**^
*Clostridium perfringens*	Cpref191	GTAGTAAGTTGGTTTCCTCG^**7**^

^1^[14]

^2^[16]

^3^[17]

^4^[18]

^5^[19]

^6^[20]

^7^[21]

### Analysis of Fermentation Products

Digesta samples were subjected to short-chain fatty acids (SCFA) analysis, using gas chromatograph (Shimadzu GC-2010, Kyoto, Japan). The samples (0.5g crop and ileum samples, 0.2g caeca sample) were mixed with 0.2 ml formic acid, diluted with deionised water and centrifuged at 7,211 × *g* for 10 min. The supernatant was loaded onto a capillary column (SGE BP21, 30 m × 0.53 mm) using an on-column injector. The initial oven temperature was 85°C and was raised to 180°C by 8°C/min and held there for 3 min. The temperatures of the flame ionisation detector and the injection port were 180°C and 85°C, respectively. The sample volume used for GC analysis was 1 μl. The putrefactive SCFA (PSCFA) concentration was calculated as the sum of iso-butyrate, iso-valerate, and valerate concentration in the digesta.

### Statistical Analysis

Statistical analysis of the results was performed using the GLM of the SAS [22] according to the following general model:

$${\text{Y}}_{ij}=\mu +\alpha_{i}+\beta_{j}+{\left(\alpha \beta \right)}_{ij}+\delta_{ij}$$

where Yij was the observed dependent variable; μ was the overall mean; αi was the effect of phytase; βj was the effect of calcium and phosphorus reduction; (αβ)ij was the interaction between phytase and calcium and phosphorus reduction; and δij was the random error. In cases where the effects were judged significant (*P*<0.05), means were compared pairwise (pdiff). Results are given as the least squares means with pooled standard error of the mean (SEM).

## Results

### Bird Performance

Effects of microbial phytase and dietary Ca and dP concentrations on the performance of broiler chickens are presented in Table 3. Mortality was low (<3%) and unrelated to the dietary treatments. In all experimental periods and for all performance parameters, no interactions between Ca and dP level and phytase supplementation were observed. There was no effect of Ca and dP concentration on feed intake (FI) throughout the experiment. However, from d 15 to 21, the reduction in dP and Ca levels decreased body weight gain (BWG) (*P*<0.001) and impaired feed conversion ratio (FCR) (*P*<0.001).

**Table 3:** Effects of the phytase and Ca/P levels on performance of broiler chickens.

	1–14 d			15–21 d			22–42 d			1–42 d		
Treatment	BWG, g	FI, g	FCR, g:g	BWG, g	FI, g	FCR, g:g	BWG, g	FI, g	FCR, g:g	BWG, g	FI, g	FCR, g:g
PC	380^b^	519	1.37^a^	424^a^	652	1.54^b^	1742	3253	1.87	2546	4423	1.74^a^
PC + PHY	406^a^	530	1.31^b^	428^a^	649	1.52^b^	1788	3228	1.81	2622	4406	1.68^b^
NC	379^b^	513	1.36^a^	399^b^	638	1.60^a^	1738	3278	1.89	2515	4428	1.76^a^
NC + PHY	407^a^	531	1.31^b^	426^a^	659	1.55^b^	1775	3270	1.85	2606	4460	1.71^a^ ^b^
Pooled SEM	3.800	3.566	0.008	3.582	4.435	0.010	12.437	18.292	0.013	16.861	22.089	0.010
Model P	0.002	0.200	0.006	0.007	0.419	0.007	0.427	0.798	0.109	0.076	0.871	0.027
Main Effects												
P and Ca level												
Optimal	393	524	1.34	426^a^	650	1.53^b^	1765	3241	1.84	2584	4415	1.71
Deficient	393	522	1.33	412^b^	649	1.58^a^	1756	3274	1.87	2561	4444	1.74
Phytase												
None	379^b^	516^b^	1.36^a^	412^b^	645	1.57^a^	1740	3266	1.88^a^	2531^b^	4426	1.75^a^
5000 FTU/kg	406^a^	531^a^	1.31^b^	427^a^	654	1.53^b^	1781	3249	1.83^b^	2614^a^	4433	1.70^b^
P-value												
P and Ca level	0.976	0.727	0.592	0.029	0.851	0.005	0.726	0.388	0.242	0.467	0.533	0.164
PHY	0.0002	0.041	0.0006	0.017	0.316	0.033	0.111	0.667	0.034	0.013	0.871	0.007
Interaction Terms												
P and Ca level x PHY	0.880	0.623	0.720	0.078	0.185	0.402	0.864	0.820	0.644	0.806	0.602	0.893

PC – positive control (diet adequate in Ca and dP levels); NC – negative control (diet with reduced Ca and dP levels by 0.14 and 0.13% respectively); PC + PHY / NC + PHY – positive/negative control supplemented with 5000 FTU of 6-phytase (EC 3.1.3.26) per 1kg of complete feed

BWG -Body Weight Gain; FER—Feed Conversion Ratio; FI—Feed Intake

SEM—standard error of the mean

^a-b^Within the same column, different superscripts indicate significant differences between treatments (*P*<0.05)

In the first period of the trial (1–14 d) the inclusion of microbial phytase at 5000 FTU/kg increased body weight gain, feed intake and improved feed conversion ratio. In the 15–21 d period, phytase supplementation also resulted in an increase in BWG and a reduction in FCR, but had no impact on feed intake. Between d 22–42 only feed conversion ratio was influenced by phytase. Overall, from d 1–42, the addition of 5000 FTU of phytase per kg of the diet resulted in an increase in BWG and a reduction in FCR, but did not affect feed intake.

### Analysis of Gastrointestinal Tract pH

The reduction in Ca and dP levels lowered pH in the caeca (Table 4). Inclusion of phytase increased pH in the crop of chicken fed the diet with an optimal Ca and dP concentrations but not in those receiving diets with a low Ca and dP levels as suggested by the significant interaction. A significant Ca and dP level x phytase interaction was also recorded for ileal pH values. The addition of phytase to the Ca and dP deficient diet (NC), resulted in an increase in ileal pH but had no impact on ileal pH in chicken fed the diet with the optimal Ca and dP concentrations (PC). Furthermore, there was no effect of phytase supplementation on the pH values in caeca.

**Table 4:** The effect of the phytase and Ca/P levels on pH in the gastrointestinal tract of broiler chicken.

	pH value		
Treatment	Crop	Ileum	Caeca
PC	6.47^b^	7.01^b^	7.36^a^
PC + PHY	6.56^a^	6.99^b^	7.22^a^ ^b^
NC	6.48^b^	6.48^c^	6.98^b^
NC + PHY	6.41^b^	7,50^a^	7.07^b^
Pooled SEM	0.013	0.073	0.049
Model P	0.0004	<.0001	0.027
Main Effects			
P and Ca level			
Optimal	6.51^a^	7.00	7.29^a^
Deficient	6.45^b^	6.99	7.02^b^
Phytase			
None	6.47	6.74^b^	7.17
5000 FTU/kg	6.49	7.24^a^	7.14
P-value			
P and Ca level	0.005	0.895	0.005
PHY	0.523	<.0001	0.775
Interaction Terms			
P and Ca level x PHY	0.0008	<.0001	0.241

PC – positive control (diet adequate in Ca and dP levels); NC – negative control (diet with reduced Ca and dP levels by 0.14 and 0.13% respectively); PC + PHY / NC + PHY – positive/negative control supplemented with 5000 FTU of 6-phytase (EC 3.1.3.26) per 1kg of complete feed

SEM—standard error of the mean

^a-c^Within the same column, different superscripts indicate significant differences between treatments (*P*<0.05)

### Microbial Community Analysis

The total number of bacteria (DAPI counts) was lowered by the reduction in Ca and P concentrations, but was increased by phytase supplementation (Table 5). None of the dietary treatments affected *Bacteroides* counts. The *Clostridium perfringens* and *Enterobacteriacae* counts were reduced in ileal digesta collected from the birds receiving the Ca and dP deficient diets. However, no direct effects of phytase addition on the above mentioned microbiota counts were observed. Both experimental factors increased the LAB counts. *Clostridium leptum* subgroup, *Clostridium coccoides*—*Eubacterium rectale* cluster, *Bifidobacterium sp*. and *Streptococcus/Lactococcus* counts were subject to a Ca and dP*phytase interaction. There were no effects of Ca and dP concentrations on the *Clostridium leptum* subgroup and the *Streptococcus/Lactococcus* counts. Phytase addition to the Ca and dP deficient diet increased the population of the *Clostridium leptum* subgroup, but had no effect when added to the diets with optimal Ca and dP level. For the *Streptococcus/Lactococcus* counts, the inclusion of phytase to the Ca and dP deficient diets had no effect but when the enzyme was added to the positive control diet it reduced the *Streptococcus/Lactococcus* population. The counts of *Clostridium coccoides*—*Eubacterium rectale* cluster were decreased by the reduction in Ca and dP concentrations. The addition of phytase to the negative control diet had no effect on numbers of this cluster of clostridia, but when added to the positive control diet the enzyme increased this population. *Bifidobacterium* counts were increased by the reduction in Ca and dP concentrations, and were decreased by phytase supplementation. The enzyme, however, exerted much more pronounced effect on the counts of *Bifidobacteria* in chickens fed the negative control diet.

**Table 5:** Effects of the phytase and Ca/P levels on ileal microbiota in broiler chickens (log cfu/ml digesta).

**Treatment**	**DAPI**	**BACTO**	**CPREF**	**ENTER**	**LAB**	**CLEPT**	**STRC**	**EREC**	**BIF**
PC	9.139^b^	7.933	8.103	8.225^a^	7.858^b^	7.604^a^ ^b^	7.656^a^	7.784^b^	7.915^a^
PC + PHY	9.592^a^	7.799	8.048	8.160^a^	8.048^a^	7.571^b^	7.492^b^	7.878^a^	7.628^c^
NC	8.572^c^	7.942	7.868	7.985^b^	8.002^a^	7.510^b^	7.567^a^ ^b^	7.703^c^	7.934^a^
NC + PHY	8.978^b^	7.911	7.961	7.894^b^	8.064^a^	7.675^a^	7.577^a^ ^b^	7.704^c^	7.748^b^
Pooled SEM	0.0467	0.0285	0.0390	0.0268	0.0200	0.0168	0.0159	0.0097	0.0169
Model P	<.0001	0.2555	0.1558	<.0001	0.0007	0.0041	0.0034	<.0001	<.0001
Main Effects									
P and Ca level									
Optimal	9.369^a^	7.865	8.075^a^	8.192^a^	7.954^b^	7.587	7.572	7.831^a^	7.769^b^
Deficient	8.775^b^	7.926	7.914^b^	7.939^b^	8.033^a^	7.593	7.572	7.704^b^	7.841^a^
Phytase									
None	8.851^b^	7.938	7.983	8.103	7.931^b^	7.556^b^	7.611^a^	7.743^b^	7.924^a^
5000 FTU/kg	9.285^a^	7.855	8.004	8.027	8.056^a^	7.623^a^	7.534^b^	7.791^a^	7.688^b^
P-value									
P and Ca level	<.0001	0.2870	0.0388	<.0001	0.0350	0.8806	0.9564	<.0001	0.0060
PHY	<.0001	0.1474	0.8069	0.1107	0.0011	0.0407	0.0129	0.0013	<.0001
Interaction Terms									
P and Ca level x PHY	0.7151	0.3679	0.3396	0.7899	0.0943	0.0025	0.0051	0.0015	0.0454

DAPI – total number of bacteria; BACTO – *Bacteroides* – Prevotella cluster; CPREF – *Clostridium perfringens*; ENTER – *Enterobacteriaceae*; LAB – *Lactobacillus sp*./*Enterococcus sp*.; CLEPT – *Clostridium leptum* subgroup; STRC – *Streptococcus/Lactococcus*; EREC – *Clostridium coccoides*—Eubacterium rectale cluster; BIF – *Bifidobacterium sp*.

PC – positive control (diet adequate in Ca and dP levels); NC – negative control (diet with reduced Ca and dP levels by 0.14 and 0.13% respectively); PC + PHY / NC + PHY – positive/negative control supplemented with 5000 FTU of 6-phytase (EC 3.1.3.26) per 1kg of complete feed

SEM—standard error of the mean

^a-c^Within the same column, different superscripts indicate significant differences between treatments (*P*<0.05)

### SCFA Concentration

The were no main effects of dietary Ca and dP levels and of phytase supplementation on the concentrations of acetic and lactic acids, as well as on the total SCFA in the crop (Table 6). However, Ca and dP level*phytase interactions were noted for acetate and total SCFA. Supplementation of the diet with reduced Ca and dP levels with phytase resulted in increased acetate and total SCFA concentrations, but when the enzyme was added to the diet with optimal levels of these minerals, the opposite effect was observed. Ileal acetate concentration, however, was reduced in chickens fed the diet with reduced Ca and dP levels. Furthermore, as noted for acetate and total SCFA concentrations in the crop, the same interaction terms were recorded for DL-lactic acid and total SCFA concentration. In the caecum, only propionic acid concentrations were influenced by the Ca and dP level*phytase interaction. There was a positive effect of phytase supplementation in the positive control diet and no effect in the negative control diet on this acid concentration (Table 7). The reduction in Ca and dP level in the diet resulted in decreased concentrations of iso-butyric and succinic acids, and also in a decreased percentage of acetate in the SCFA profile. However, the concentration of n-butyric acid as well as its percentage of SCFA were increased in the caeca digests collected from chickens fed the diets deficient in Ca and dP. Phytase addition, on the other hand, affected only the proportions of acetate in the SCFA profile, reducing them by 2%.

**Table 6:** Effects of the phytase and Ca/P levels on SCFA concentration in crop and ileal digesta (mmol/kg digesta).

	Crop			Ileum		
Treatment	Acetic acid	DL-Lactic acid	Total SCFA	Acetic acid	DL-Lactic acid	Total SCFA
PC	4.25^a^	3.97	8.23^a^	6.475^a^	6.99^a^	13.47^a^
PC + PHY	3.64^a^ ^b^	1.59	5.23^b^	5.78^a^	5.32^b^	11.10^a^ ^b^
NC	3.35^b^	1.24	4.59^b^	3.47^b^	5.53^b^	9.12^b^
NC + PHY	4.40^a^	4.36	8.76^a^	5.23^a^ ^b^	6.52^a^ ^b^	11.75^a^ ^b^
Pooled SEM	0.149	0.750	0.857	0.401	0.250	0.547
Model P	0.029	0.342	0.022	0.044	0.046	0.035
Main Effects						
P and Ca level						
Optimal	3.95	2.78	6.73	6.13^a^	6.16	12.28
Deficient	3.88	2.80	6.68	4.35^b^	6.03	10.43
Phytase						
None	3.80	2.60	6.41	4.97	6.26	11.29
5000 FTU/kg	4.02	2.97	6.99	5.50	5.92	11.42
P-value						
P and Ca level	0.800	0.990	0.976	0.022	0.777	0.072
PHY	0.422	0.806	0.727	0.476	0.462	0.898
Interaction Terms						
P and Ca level x PHY	0.004	0.075	0.040	0.105	0.007	0.018

PC – positive control (diet adequate in Ca and dP levels); NC – negative control (diet with reduced Ca and dP levels by 0.14 and 0.13% respectively); PC + PHY / NC + PHY – positive/negative control supplemented with 5000 FTU of 6-phytase (EC 3.1.3.26) per 1kg of complete feed

SEM—standard error of the mean

^a-b^Within the same column, different superscripts indicate significant differences between treatments (*P*<0.05)

**Table 7:** Effects of the phytase and Ca/P levels on SCFA concentration and profiles in caeca digesta (mmol/kg digesta).

Treatment	Acetic acid	Propionic acid	Iso-butyric acid	n-Butyric acid	n-Valeric acid	Succinic acid	Total SCFA	%acetic	%propionic	%butyric
PC	49.46	4.29^b^	1.04^a^	5.78	1.24	7.17	69.41	82.78^a^	8.54	8.69
PC + PHY	46.10	6.52^a^	0.91^b^ ^a^	5.22	1.24	4.59	64.58	79.96^a^ ^b^	11.16	8.88
NC	53.49	6.68^a^	0.76^c^ ^b^	7.63	1.39	2.74	72.68	79.04^b^	9.85	11.12
NC + PHY	48.16	5.60^a^ ^b^	0.71^c^	8.50	1.33	1.73	66.03	77.63^b^	9.16	13.21
Pooled SEM	1.809	0.333	0.040	0.589	0.060	0.794	2.199	0.575	0.554	0.678
Model P	0.548	0.034	0.006	0.161	0.773	0.071	0.586	0.007	0.392	0.051
Main Effects										
P and Ca level										
Optimal	47.78	5.41	0.98^a^	5.50^b^	1.24	5.88^a^	66.99	81.37^a^	9.85	8.78^b^
Deficient	50.83	6.14	0.73^b^	8.07^a^	1.36	2.23^b^	69.36	78.33^b^	9.50	12.17^a^
Phytase										
None	51.48	5.49	0.90	6.70	1.31	4.95	71.04	80.91^a^	9.19	9.90
5000 FTU/kg	47.13	6.06	0.81	6.86	1.28	3.16	65.30	78.80^b^	10.16	11.05
P-value										
P and Ca level	0.414	0.234	0.001	0.031	0.324	0.020	0.602	0.004	0.756	0.011
PHY	0.246	0.349	0.195	0.892	0.808	0.236	0.210	0.039	0.389	0.366
Interaction Terms										
P and Ca level x PHY	0.790	0.010	0.562	0.533	0.824	0.598	0.840	0.479	0.146	0.450

PC – positive control (diet adequate in Ca and dP levels); NC – negative control (diet with reduced Ca and dP levels by 0.14 and 0.13% respectively); PC + PHY / NC + PHY – positive/negative control supplemented with 5000 FTU of 6-phytase (EC 3.1.3.26) per 1kg of complete feed

SEM—standard error of the mean

^a-c^Within the same column, different superscripts indicate significant differences between treatments (*P*<0.05)

## Discussion

The negative control diet was formulated to contain around 0.13–0.14% less Ca and dP than the positive control diet and it was expected that this would cause a decrease in both BWG, and FI. Clearly, this was not the case suggesting that the deficiency was small and, indeed, a depression in BWG and FCR was only noted from 15 to 21 d only. One possible reason for the lack of a deficiency may be due to the fact that phytate-P digestibility, in the absence of phytase, can be very high, especially if Ca levels are low [23, 24]. Indeed if dietary Ca concentration is reduced to around 0.2% it phytate P digestibility can reach 80%. It is thought that this may be due to reduced interaction between phytate and Ca, allowing for a greater efficacy of phytases derived from feed ingredients, the brush border and the microbiota of the host, and through the reduction in the formation of insoluble Ca-phosphate complexes [23, 25]. These findings illustrate an important point, that if dietary Ca concentrations are reduced then dietary digestible P concentrations will increase irrespective of presence or absence of other intervention strategies [24, 26]. Moreover, the reduction in dietary Ca may also indirectly improve amino acid (AA) utilization by facilitating a decrease in gastric pH and subsequent improvement in pepsin efficacy [8]. In the present study dietary Ca concentrations were reduced by 0.14%. Any negative effects of this reduction were most likely muted by enhanced digestibility of phytate P. Such effects have been noted elsewhere. Indeed, Walk et al. [27] reported no deterioration in FI, FCR or BWG when 42 or 49-d-old broilers were fed corn-soybean diets which were apparently deficient in Ca and available P by 0.16%, and 0.15% respectively, as compared with the positive control diet. In that study, however, younger birds (0–21 d) did exhibit reduced BWG without changes in FI and FCR, suggesting that young chickens may be more susceptible to a marginal P deficiency than older birds [27]. However, in our studies, there were no differences in BWG and FCR between the positive and negative controls from 1–14 d and 1–42 d of age, suggesting Ca and P were not limiting in either diet. The fact that phytase supplementation improved both BWG and FCR on both of these diets over the same period suggests that this effect is not mediated through incremental Ca and/or P availability. It was only in the 15–21 d experimental period, that the data suggest the negative control was Ca and/or P limiting and thus only in thes period could the improvements in BWG and FCR been due to Ca and/or P release. Overall, however, the benefits recorded in BWG and FCR on feeding the phtyase in the first and last periods, and also when calculated over the entire experiment, may be related to improved utilization of amino acids and energy [28]. Phytate content of the finisher diets was certainly higher than in the starter diets due to the higher inclusion rate of rapeseed expeller which likely allowed for a greater response to phytase. Phytate is one of the main antinutritional components in rapeseed and its byproducts, with the content approximating 3.3% in rapeseed expeller meal [29–32]. Indeed Smulikowska et al. [30] found that phytase supplementation of rapeseed cake supplemented diets increased apparent total tract protein digestibility confirming the negative effect of phytate on AA retention. In the same study, however there was no effect on total P retention suggesting the effect on AA retention is not related to P release but probably phytate destruction.

Hydrogen ion concentration varied significantly among specific sections of the digestive tract of the chicken and between individuals as well. The pH of the digestive tract can be influenced by many factors including Ca and P concentration of the diet. It is well documented that high levels of Ca affect pH in certain parts of the digestive tract [7]. In the present study, the reduction of Ca and P concentration in a diet was associated with decreases in pH values measured in the caeca. Limestone and dicalcium phosphate have a very high acid-binding/buffering capacity and contribute to an increase in digesta pH [33]. Thus, the higher caecal pH of chickens fed the positive control diet may have been directly attributable to its greater Ca content. Guinotte et al. [34] found that an increase in dietary Ca from 1 to 3.6% increased pH from 2.76 to 3.82 in the gizzards of pullets. Similarly, Walk et al. [8] observed a reduction in gizzard and ileal pH values in broilers fed 0.64% Ca as compared with those fed 1.03%. In contrast, Smulikowska et al. [32] did not find any effect of increasing the monocalcium phosphate content of the diet on the pH of the crop, stomach, and in ileal and caecal digesta. Care must be taken, however, to differentiate between calcium coming from monocalcium phosphate and that from limestone, as the latter provides a direct buffer and does not provide an acidic anion as does the former. From a physiological point of view, therefore, limestone may be a more important buffer than monocalcium phosphate.

The two main experimental factors, phytase and dP and Ca levels interacted for pH values in the crop and ileum. Addition of phytase raised pH of the PC such that it was significantly greater than all other treatments. The scale of this response was small however compared with the effects noted in the ileum where was no effect of phytase on pH of the PC birds, and the NC birds had a lower ileal pH than all other diets. Again this may reflect the higher buffering capacity of the PC diets. Addition of phytase to the NC resulted in a large (1 pH unit) increase in ileal pH, such that it was significantly higher than all other treatments. Application of phytase would be expected to ease the constraints on gastric digestion such that inputs of HCl and pepsin can be reduced without compromising digestion and thus pH rises, as has been noted previously [27]. This would reduce the H ion content of the digesta effluxing from the gizzard and hence enable a more complete neutralisation of the digest—hence the elevated pH in the ileum. The inconsistency which cannot be explained is why such an increase in ileal pH was not noted in the PC when it was supplemented with phytase. The literature is also not consistent in this regard. Applying phytase at 1,000 FTU/kg to diets with high and low levels of P, Smulikowska et al. [32] observed an increase in the pH of jejunal digesta, while pH of the crop, stomach, ileal and caecal digesta were not affected. Furthermore, the application of a microbial phytase did not influence gastrointestinal pH (in the crop, gizzard, duodenum, jejunum, ileum) in 42-d-old broilers [35] or stomach pH values in weanling pigs [3]. Supplemental phytase, however, increased fecal pH but did not affect ileal pH in pigs [1]. Also Akyurek et al. [36] found an increase in caecal pH but not in ileal pH in 21-d-old broilers when microbial phytase was incorporated at 500 FTU/kg of a reduced P diet. Walk et al. [8] reported that feeding 5,000 FTU/kg significantly increased gizzard, duodenum, jejunum and ileum pHs in 16-d-old broilers, and that this effect was consistent in a low and a high Ca diets. Furthermore, Walk et al. [8] suggested that the increase in the gastrointestinal pH created by high levels of supplemental phytase may directly arise from the breakdown of the phytate molecule, release of Ca, and from a reduction of the acidogenic activity of phytate. The fact that there are so many conflicting reports regarding the effects of phytase and Ca:P levels on the gastrointestinal pH values in monogastrics may be partly explained by different phosphate sources, different levels of inclusion of Ca and P, and different animal ages used by research teams discussed above.

The amorphous calcium phosphate complex can also influence fermentation processes in the gastrointestinal tract. In rats and ruminants it has been shown that Ca and P are important modulators of microbial fermentation [37, 38]. For example, there is a minimal P requirement for the fermentation of carbohydrates by rumen bacteria [39]. According to *in vitro* studies, depletion of P reduced the fermentative activity of rumen bacteria, resulting in decreased production of SCFA and reduced bacterial ATP concentrations[39]. In the present study, the interaction between phytase and dietary Ca and dP was manifested by the fact that reducing the Ca:P content of the diet reduced total SCFA, DL-lactate and acetic acid in the ileum and phytase increased concentrations of these acids only in broilers fed the NC diet. This suggests that phosphate supply may well have been a limiting factor for fermentation in the ileum. Similar observations were made for the total SCFA content of the of crop. Smulikowska et al. [32] found that total SCFA as well as acetic acid, propionic acid and butyric acid concentrations in the caecal digesta of 29-day-old chickens were increased with increasing dietary phosphorus content. Moreover, Smulikowska et al. [32] also found that phytase supplementation increased acetic acid and, butyric acid concentrations but only in the diet supplemented with the lower and not the higher monocalcium phosphate inclusion level, suggesting a maximum threshold had been reached. In contrast, in the study of Metzler et al. [1] supplemental monocalcium phosphate did not significantly increase ileal or fecal SCFA concentrations in pigs but it did significantly reduce ileal DL-lactate concentrations. Further, Metzler-Zebeli et al. [40] demonstrated that high-dietary monocalcium phosphate decreased total lactate and L-lactate concentrations in the gastric digesta of pigs. In the same study, however, phytase supplementation did not affect ileal concentrations of SCFA and lactate, but reduced fecal concentration of n-butyrate and its molar proportion. However, in later work, Metzler-Zebeli et al. [41] found no effect of dietary monocalcium phosphate on ileal SCFA concentrations. In this regard, the data relating SCFA concentrations in the gastrointestinal tract of monogastrics to phosphate supply are equivocal, likely for the same reasons as discussed for the pH data above.

The SCFA play an essential role as bioregulators and mucosal growth promoters via direct or indirect mechanisms in the gut, as well influencing metabolism systemically once absorbed into the blood [42, 43]. It may be expected that the changes noted in the current study in SCFA concentrations are the result of changes in the microbial population or activity. It has been shown in rats that high dietary Ca and P content increased intestinal colony numbers of lactobacilli [44]. In other work a diet rich in dietary Ca and P resulted in decreased numbers of *Enterococcus* spp., *Clostridium* cluster IV and lactobacilli species in the ileum of growing pigs [40]. Further work noted reduced counts of *Streptococcus* spp. in the stomach of pigs fed high Ca and P diets [41]. However, in this latter work, *Clostridium* cluster XIVa numbers were increased by the high Ca:P diet. These findings are in agreement with the study reported here, in which the diets deficient in Ca and dP reduced butyrate but increased lactate producing bacterial groups. Additionally, in the present study, a reduction in *Clostridium perfringens* and *Enterobacteriaceae* counts were also shown with low Ca:P diets. Calcium can influence bacterial growth by a range of factors, such as counteracting the adverse effects of acidic fermentation and precipitating cytotoxic components within the intestinal lumen [44], induction of increased gastric acid secretion [45] or interference with bacterial adhesion to the intestinal mucosal walls [46, 47].

The addition of phytase increased the total numbers of bacteria, as well as *Lactobacillus sp*./*Enterococcus sp*. Enzyme supplementation increased *Clostridium leptum* numbers only in the NC and *Clostridium coccoides* only in the PC subgroups. On the other hand, the counts of *Streptococcus/Lactococcus* and *Bifidobacterium sp*. were reduced in broilers fed the PC diets supplemented with phytase The reduction in *Bifidobacterium sp*. counts was more profound, however, in chicken fed the NC diet. Metzler-Zebeli et al. [40] also found that addition of phytase increased the ileal numbers of strictly anaerobic bacteria, such as the *C*. *coccoides* cluster, the *C*. *leptum* cluster, and the *Bacteroides*-*Prevotella*-*Porphyrmonas* group, and tended to enhance those of *Enterobacteriaceae*. The authors stated that these findings might be associated with the greater availability of P in the lumen of the gastrointestinal tract. Based on the data reported here we must emphasize that such effects of phytase are influenced strongly by Ca and P content of a diet and that excessive P supply, perhaps achieved by the combination of the high Ca:P diet with phytase addition, may negatively influence some bacterial populations.

Phosphorus supply may indeed play a controlling role for the total numbers of bacteria as suggested by the significant Ca/P and phytase effects which were independent of one another, and tended to be additive. Phytase seems to have an important role to play in modulating the gut flora but their effects are clearly framed by the background levels of P and Ca in the diet. The changes in the microbiome noted in this paper as a result of feeding a phytase at such high inclusion levels are novel, although it must be noted that although the changes were significant, the scale of the changes were not extraordinary. The fact that phytase, rather than Ca and P levels played a significant role in promoting better animal performance suggests that the linkage between microbiome structure and performance is not inextricable.
